# Application of Optical Coherence Tomography Angiography to Retinal Disease

**DOI:** 10.1155/2018/5490592

**Published:** 2018-08-07

**Authors:** Talisa E. de Carlo, Nadia K. Waheed, Jayanth Sridhar, Daniela Ferrara

**Affiliations:** ^1^Illinois Eye and Ear Infirmary, University of Illinois at Chicago, Chicago, IL, USA; ^2^New England Eye Center, Tufts University School of Medicine, Boston, MA, USA; ^3^Bascom Palmer Eye Institute, University of Miami, Miami, FL, USA

A little over two years ago, a hot new topic in retinal imaging caught the attention of ophthalmologists around the world. Optical coherence tomography angiography (OCTA), an innovative, fast, noninvasive, non-dye-based angiographic technique, quickly became a topic of great interest in major conferences and ophthalmology journals. Inquisitive minds applied the OCTA prototypes to understanding diseases such as macular degeneration, diabetic retinopathy, and glaucoma. The technology's high resolution and ability to segment the different vascular layers provided novel insight into disease pathogenesis and morphology, providing a unique opportunity to investigate the ocular microvasculature in a level of anatomic detail that is not achievable with any other imaging strategy.

However, as with any new technology, OCTA has limitations that should be taken into account when interpreting its results, in both the research and clinical settings. Image artifacts and the inability to accurately quantify vascular parameters are some of the broadly recognized limitations, in addition to others related to variable segmentation algorithms and to the physics of image acquisition and processing. Today, advancements in software that address some of the limitations and more widespread access to the OCTA devices allow investigators to further expand upon our collective knowledge of ophthalmologic disease. In this special issue, we compiled a collection of high-quality research articles to discuss the state-of-the-art application of OCTA to chorioretinal diseases.

Five papers are included in this special edition that focus on a variety of different diseases and innovative applications of OCTA. One of these papers is a review of the choriocapillaris changes seen in advanced age-related macular degeneration (AMD) noting that OCTA has contributed significantly to our knowledge of the development and progression of geographic atrophy and choroidal neovascularization (CNV). The manuscript highlights that choriocapillaris atrophy occurs underneath and beyond the region of photoreceptor and retinal pigment epithelium loss and that CNV seems to originate from these areas of choriocapillaris alterations. A second paper included in this special edition also utilizes OCTA to study AMD but focuses on its value at the outer retina. The paper explores the changes seen in CNV after antivascular endothelial growth factor in treatment-naïve eyes compared with previously treated eyes. Qualitatively, the two groups respond similarly to treatment, but, quantitatively, the treatment-naïve eyes demonstrate a statistically significant decrease in CNV size while there is no difference in CNV size in the eyes that were previously treated.

A third paper focuses on the use of OCTA in detecting silent type 1 CNV in patients with chronic central serous chorioretinopathy. The authors show that OCTA delineates CNV in 8.3% of the treatment-naïve eyes in which OCT and fluorescein angiography (FA) did not show a CNV. A fourth paper compares the sensitivity and specificity of detection of polypoidal choroidal vasculopathy (PCV) using OCT and FA together with the sensitivity and specificity of OCT, FA, and OCTA together. The work demonstrates that specificity is not improved when adding OCTA to the arsenal but sensitivity of PCV detection is enhanced from 69.5% to 90%. Lastly, the fifth paper employs the flow density function of OCTA to prospectively compare macular and optic nerve head vascular flow before and after cataract surgery plus implantation of two iStents and before and after cataract surgery alone. The authors demonstrate that there is statistically improved flow density in the macula and optic nerve head after cataract surgery plus iStent implantation but no change in eyes that underwent cataract surgery alone. These five manuscripts demonstrate interesting new applications of OCTA, contributing even further to both our understanding of the new technology and to the chorioretinal vascular diseases that are under investigation. As the scientific community moves forward with additional technological advances on OCTA, clinicians come together to establish new treatment paradigms based on this revolutionary imaging modality.

*Talisa E. de Carlo* *Nadia K. Waheed* *Jayanth Sridhar* *Daniela Ferrara*

